# Increased Comfort of Polyester Fabrics

**DOI:** 10.3390/polym13173010

**Published:** 2021-09-06

**Authors:** Meritxell Martí, Jaime Gisbert-Paya, Mª Ángeles Bonet-Aracil, Petar Jovančić, Manuel J. Lis, Luisa Coderch

**Affiliations:** 1 Institute for Advanced Chemistry of Catalonia (IQAC-CSIC), 08034 Barcelona, Spain; luisa.coderch@iqac.csic.es; 2 Escuela Politécnica Superior de Alcoy, Universitat Politècnica de València (UPV), 46022 Alcoy, Spain; jaigispa@txp.upv.es (J.G.-P.); maboar@txp.upv.es (M.Á.B.-A.); 3 Centre Tecnológic de Catalunya (EURECAT), Unitat de Teixits Funcionals, 08302 Mataró, Spain; petar.jovancic@eurecat.org; 4 INTEXTER-UPC, 08222 Terrassa, Spain; manuel-jose.lis@upc.edu

**Keywords:** comfort, polyester, glycerol, moisture content, DVS, water vapor resistance

## Abstract

The hydrophilicity of fibers is directly related to the comfort of a fabric and represents one of the most important aspects of a textile. Therefore, polyester (PES) modification has focused on an increase in moisture content and a subsequent improvement of the user’s experience. Based on the glycerol hygroscopic properties, the main objective has been the enhancement of the hydrophilicity of polyester by glycerol treatments. Furthermore, microwave irradiation and alkaline treatment have been applied, in order to increase glycerol adhesion. Treated PES samples were characterized by performing moisture content, negative ion, water diffusion and water vapor resistance analyses. The effect of different treatment conditions such as bath ratio (1/10 or 1/15), temperature (40, 60 or 100 °C), time (2 or 5 min) and microwave radiation intensity (300 or 500 W) was evaluated. The moisture content of treated PES results indicated that by decreasing the bath ratio and increasing the time and temperature the moisture gain can reach almost 14%, which can be easily related to increases in the weight of the fiber. The treatment with alkali was done and led to the highest moisture increase. Treatment with 500 W microwave irradiation led to higher glycerol retention after rinsing. Different experimental conditions were applied to the glycerol-treated PES fabrics, and a clear improvement in moisture content was obtained increasing the comfort. The results were compared with the ones obtained for cotton and wool, where the moisture is higher than non treated PES.

## 1. Introduction

The thermophysiological comfort of a garment is determined by the fabric’s air permeability (AP), moisture management and heat transfer properties [1,2]. The removal of unnecessary heat and moisture from the body helps to improve the comfort level. Therefore, in warm and humid environments, higher fabric AP increases comfort [3], which indicates the psychological and physical harmony of humans with their microclimate [4]. If perspiration is trapped next to the skin during physical activity, then body temperature may increase and lead to dehydration, fatigue and decreased performance [4,5]. The comfort properties of garments depend on the balance between moisture absorption and the sweat wicking capacity of fabrics, and these characteristics are associated with the fabric’s structure, composition and processing. The fabric can be considered a buffer as it absorbs moisture and wicks sweat, thereby transporting moisture away from the body and facilitating evaporation from the outside of the fabric. The cooling caused by evaporation contributes to the wearer feeling more comfortable. Sweat wicking capacity can be determined by assessing the wettability of the fabric. Sweat reaches the capillaries, and the developed pressure forces the sweat to move along the capillaries, resulting in a certain amount of liquid transferred into the fabric [6].

Therefore, an ideal fabric possesses a high thermal resistance for protection from cold weather, a low water vapor resistance for efficient heat transfer under soft thermal stress conditions and a rapid liquid transport characteristic for transferring heat and eliminating unpleasant tactile sensations [7].

Therefore, the hydrophilicity of fibers is directly related to comfort, which is one of the most important aspects of textile fabrics. Therefore, one of the objectives of this work is to increase the hydrophilicity of polyester fabrics (PES). Specifically, the importance of fabric transpiration is emphasized since this property is directly linked to the comfortable use of garments. The evaporation of moisture due to sweating should not change the body temperature [8]. PES fabrics are easy to care for and dry quickly, but they do not perspire and even promote/accumulate sweating at elevated temperatures to a greater degree than cotton.

Moreover, the hydrophobic properties of a fiber are also well known to affect its antistatic and antisoiling properties. Hydrophobic fibers, when formed into shaped textile articles, tend to accumulate static charges with a propensity to accept and retain grime and dirt. Synthetic hydrophobic fibers also tend to be oleophilic. Therefore, should oil and grime become embedded in the fiber, the hydrophobic properties tend to prevent water from entering the fiber to remove contaminants. Given the difficult problem of cleaning oleophilic fibers, these properties should be modified to permit the entrance of water for ease of cleaning [9].

Taking into account that comfort is not just the moisture content or hydrophilicity, this study also focuses on water vapor resistance and on increasing well-being and comfort through the generation of negative ions in the fabric. Fabrics with hydrophilic properties and/or microencapsulated materials, which are capable of generating and releasing negative ions based on their piezoelectricity and pyroelectricity [10], can take advantage of the friction of the textile with the body to enhance the generation of negative ions and consequently enhance the positive sensation of the fabric. Ions greatly influence our biological and mental processes. Negative ions in the air are able to evoke a wide range of responses in humans and may be able to influence mood, behavior and performance of certain tasks [11]. Therefore, the PES modifications will also focus on increasing the presence of negative ions and the consequent improvement in the user’s well-being.

PES fiber has a moisture content of approximately 0.4%, and thus, a very low comfort level. PES manufacturers are developing PESs by surface modification to improve the moisture absorption and wicking properties. Altering the surface characteristics of PES is rather difficult due to its inactive chemical structure. However, modification of the PES surface has been reported using various techniques, such as chemical introduction of sugars onto PES fabric using cyanuric chloride [12], protein immobilization on PES film [13], application of silk sericin [14] and ciclodextrin-based finishes for PES fabric [15]. Treatments of PES with polyethylene glycol and metal hydroxide have been reported [16]. In addition, biodegradable polymers, such as polyvinyl alcohol, have been used to bind to the PES surface in alkaline media [17]. Treating PES fibers with alkali under controlled conditions has become a common industrial practice [18]. In addition, the use of ethylene glycol and glycerin to replace the conventional water treatment as the solvent of alkaline solution has been applied to shorten the hydrolysis treatment time and increase the hydrophilicity and dyeability of PES [19]. A novel green approach for dyeing PES has been presented to minimize water consumption using a glycerin-based eutectic solvent as a dyeing medium [20].

Based on the properties of glycerol as a solvent, thickener, dispersant and hygroscopic agent used in textile processing, the promotion of the hydrophilicity of PES to improve the comfort of the fiber has been attempted. Alkaline solution has been applied to improve the tactility and activate the fiber to increase the wash fastness of glycerol. Moreover, a low energy and environmentally friendly methodology, using microwave irradiation, has also been applied. Microwave irradiation has been widely used as a special heating method in different textile processes, such as dyeing and finishing and to promote hydrolysis on polyester fabrics [21], and it has been shown that the microwave dyeing method is better than the conventional method due to the shorter dyeing time, energy savings and better dye uptake [22,23]. Microwave heating is quite different from conventional heating, where heat must diffuse into the media from the surface of the material. In volumetric heating, the materials can absorb microwave energy directly and internally and convert it into heat, which leads to advantages, such as rapid, controlled, selective and uniform heating [24]. Moreover, it is known that microwave heating enhances the diffusion of organic molecules in polymers, which can increase the fixation rate of dyes in polymeric textiles [25].

Therefore, the PES samples treated with glycerol under differential experimental conditions were characterized in terms of moisture retention and wicking properties, negative ion presence, water diffusion and water vapor resistance performance. The different treatment conditions, including the bath ratio, temperature, time and microwave radiation intensity, were evaluated and found to be related to the comfort of the textiles.

## 2. Materials and Methods

The fabrics were plain woven polyester (Style 700-480, polyester poplin, 150 g/m^2^), plain cotton fabric (CO) (bleached and desized cotton print cloth, Style 400, ISO 105-F02, 100 g/m^2^) and knitted wool (WO) (chemically bleached, 470 g/m^2^) provided by Lumaquin S.A. (Barcelona, Spain).

Glycerol (GL) was supplied by Sigma-Aldrich (Madrid, Spain), and NaOH was supplied by Carlo Ebra (Milan, Italy).

All fabric samples were conditioned under standard atmospheric pressure at 20 ± 2 °C and 65 ± 5% relative humidity (ISO 554-1976) for 24 h in a climatic chamber (CM-0/48, Dycometal, Viladecans, Spain) prior to application. GL solutions were prepared by mixing the GL with distilled water following bath exhaustion proportions to generate 3%, 50% and 100% owf (over weight of fibre) of GL related to the fabric. The application of the GL solution to the fabrics was performed by a bath exhaustion process using a GLF1083 shaking water bath (Gesellschft für Labortechnik mbH, Burgwedel, Germany). Alkaline treatments were performed following the same methodology, although the bath was 1 M NaOH solution instead of distilled water. An Owen Samsung TDS (UK) microwave was used to irradiate the fabrics at different intensities (300 and 500 W) and times (2 and 5 min) using the same GL solutions as the bath exhaustion application.

Each process was performed in triplicate. The treated fabric samples were finally dried and conditioned at 20 ± 2 °C and 65 ± 5% relative humidity for 24 h before weighing and performing the subsequent experiments. After treatment, some samples were rinsed to prove the rapidity, with 6 g of treated PES rinsed three times in the same distilled water bath (bath ratio, BR: 1/50), and dried at room temperature.

The moisture content was evaluated in the nontreated and treated fabric samples. A sample of 0.5 g was maintained in a conditioned room (20 ± 2 °C and 65 ± 5% RH) for at least 24 h before being weighed and subsequently dried in an oven at 105 °C for 24 h. After the sample was cooled in a desiccator under a P_2_O_5_ atmosphere, it was weighed again and the moisture content was calculated as a percentage in triplicate.

In the vertical wicking test, a sample of 15 mm × 100 mm was suspended vertically in an Erlenmeyer flask with 10 mm of the solution containing 2.298 g/L NaCl and 0.368 g/L CaCl_2_ [26], and the lower end of the sample was immersed in 10 mm of the liquid. The length reached in 60 s was recorded. Three specimens of each sample were tested.

The infrared spectra of the textile samples were obtained using an Avatar 360-FT-IR spectrophotometer (Thermo Fisher Scientific, Waltham, MA, USA) equipped with a Smart iTR Attenuated Total Reflectance (ATR) sampling accessory, which used a diamond crystal with a 42° incident angle. The same conditions were used for all analyzed spectra: 32 scans, resolution of 4 cm^−1^, and wavenumber range of 4000–525 cm^−1^. The analysis of the spectra was performed using OMNIC software version 8.1.210 (Nicolet, Madison, WI, USA).

Dynamic vapor sorption (DVS) measures differences in the humidity content and the water diffusion/velocity when exchanging water with the environment. DVS was performed using a thermogravimetric balance Q5000SA Sorption Analyzer (TA Instruments, New Castle, DE, USA) with a controlled humidity chamber to measure the vapor absorption and desorption. Experiments were conducted using 10 ± 1 mg of sample with a total gas flow of 200 mL/min at 25 °C according to a previously described procedure [27].

Regain measures the capacity of the fiber to absorb water (water uptake vs. the bone-dry mass of the sample), and the humidity content at 95% relative humidity (Rg95%) represents the maximal amount of water which can be absorbed by the fiber. The method applied by Vickerstaff [28] to study the diffusion of dyes within fibers was used to determine the diffusion coefficient and is represented by an expression derived from Fick’s equation that is applied to moisture diffusion. This expression yielded satisfactory results for the early stages of moisture absorption, as in the case of dye diffusion. If the fraction of absorbed water is plotted against the square root of the absorption time, the points should lie on a straight line:

(1) $$R(t)/R_{f}=\sqrt{D_{A}}\sqrt{t}$$

The slope is considered to be the square root of the apparent diffusion coefficient, *D_A_*, of the water. If the apparent diffusion coefficient is measured over the sample’s mass instead of the sample’s surface, it is measured in min^−1^.

Water vapor resistance (R_et_) was measured using a Permetest instrument (Sensora, Czech Republic) according to the ISO 11092 standard under laboratory conditions (T: 21 ± 2 °C, RH: 50 ± 2%). This instrument permits the nondestructive determination of water vapor and the thermal resistance of textile fabrics. A 12 cm × 12 cm sample was placed in the Permetest instrument, and R_et_ values were recorded in triplicate for the treated and untreated samples.

Negative ionization of the air was measured with the Air Ion Countermeter COM-3200Pro model of the COM System (Tokyo, Japan) to determine the amount of ions released from the textile. This system allows for quantification of the number of ions present in the air [29]. To facilitate the release of ions from the textile during the test, the fabric is subjected to slight automatic friction by fixing it on a magnetic stirrer and moving a magnet with a cylindrical body of 3 cm in length. For 15 min, the fabric is subjected to the movement of the magnet at 200 rpm, and the average value for 15 min is calculated, this process is performed on 3 different zones of the sample to obtain the arithmetic average. These conditions have been established as optimal after performing various tests [29,30,31].

## 3. Results and Discussion

First, the moisture content and other comfort properties of PES were assessed and compared with that of cotton (CO) and wool knitted fabric (WO), which are known to be more comfortable than PES (Table 1). Therefore, further glycerol treatments of the PES fabric (GL/PES modification) were performed with the purpose of providing comfort to the fiber in a manner similar to the one from natural fibers.

The moisture content of the PES, CO and WO were evaluated gravimetrically at 65% RH, and they presented 0.5, 5 and 10% moisture. This huge difference could be correlated with the greater importance of the negative ionization of the air from the natural fibers than for PES, although CO produces many negative ions. The moisture content of approximately 0.5% and only 1 negative ion/cm^3^ for PES were very low values, which rendered the PES fiber uncomfortable. The DVS was also evaluated, and differences in the moisture content and the water diffusion/velocity were measured when exchanging water with the environment. While the moisture at 95% HR only increased to 0.6% for PES, the moisture content reached 14% for CO and 22% for WO. In addition, the apparent diffusion results indicated a speed of water diffusion at least ten times higher in the case of PES. These results are also in agreement with the R_et_ values obtained using the Permetest water vapor resistance measurement. Therefore, although PES has a low resistance to water vapor, meaning that it is highly breathable, CO has somewhat lower breathability and WO has even less. The R_et_ values are clearly related to D_A_ by DVS.

To increase the hydrophilicity of the PES fibers and positively impact the comfort properties, different treatments with GL were performed. PES was subjected to 1/15 BR to three different GL concentrations in water (2.0, 33.3 and 66.6 mg/mL), and this process accounted for 3%, 50% and 100% of the GL related to the PES fiber. Those treatments were performed at 40 °C for 1 h (Treatments 1–3 Table 2). After these three treatments, the fiber weight increased to 9% owf, which could be due to the low percentage of GL in the fiber and the moisture content, which was also evaluated. Most importantly, the moisture content of the fiber also increased to 9% for the treatment with the highest amount of GL, i.e., 100% owf (over weight of fiber). It is important to highlight the great increase, which was even higher than the one from the corresponding CO sample. The treated textiles were then rinsed three times, as detailed in the experimental section, to determine the wash fastness. An increase in the weight of the fabrics remained. However, it accounted for only 1.9% of the PES 1/15 GL-100 treatment at 40 °C for 1 h.

Then, the bath ratio, temperature and treatment time were modified to increase the moisture content and wash fastness of PES. The bath ratio was decreased to 1/10 (treatments 4 to 11), the temperature was increased to 60 °C (treatments 8–11), and the time of treatment was increased to 3 h (treatments 6, 7, 10 and 11). The treatments maintained 50 and 100% GL related to the PES fiber (Table 2). A comparison between treatments 2 and 3 with 4 and 5 indicated a 30% moisture gain due to the decrease in the bath ratio to 1/10. A comparison between treatments 4 and 5 with 8 and 9 and between treatments 6 and 7 with 10 and 11 indicated a moisture gain ranging from 2 to 45% depending on the GL concentration due to the temperature. A comparison between treatments 4, 5, 8, and 9 with treatments 6, 7, 10, and 11, respectively, also demonstrated an increase in moisture content ranging from 15 to 30% due to the treatment time. In all cases, the change of experimental conditions, namely, the decreased bath ratio and increased time and temperature, led to an increase of the moisture content to almost 14%, which can be easily related to the weight increase of the fiber.

The wash speed of GL was also evaluated to demonstrate its fixation onto the fiber. The textiles were also rinsed, and the weight of the textile after treatment and rinsing diminished to approximately 30%. However, this value was approximately 4–7% in some cases. This important change indicates that an increase in the hydrophilicity of the fiber occurred. Moreover, in addition to the bath ratio and temperature, longer treatments promoted the highest fixation.

However, a bath ratio of 1/10 is too low to reach a homogeneous treatment at the lab level, where the sample is folded and the 3 h treatment is too long for practical use. Therefore, a higher temperature of 100 °C was assayed with 100% GL to determine the effects on the fiber (treatment 12). For treatment 3 at 40° 1 h, the moisture content is improved; however treatments with a bath ratio of 1/10 and 3 h of treatment (e.g., 5, 7, 9 and 11) presented higher moisture contents and higher fixation. To improve the fixation between the PES fabric and GL, treatments that could affect PES fibers by altering its hydrophobicity and generating some functional groups on its surface were searched. Strong alkaline treatments under high processing temperatures were assayed, since it was demonstrated the promotion of hydrophilicity and improving the comfort characteristics [17]. Hydrolytic scission of ester linkages of the polyester chains on the fibre surface takes place, providing sites for possible glycerol reaction [32,33]. In addition, microwave irradiation was also used. 

Therefore, alkaline treatments and microwave treatments (Table 3) were performed with GL at the two concentrations previously assayed. The moisture content was evaluated, not only after the different treatments, but also after the treatments and after rinsing to validate fixation. Treatments 11 and 12 were previously identified as the best, and they were also analyzed to determine the moisture content after rinsing and compared with the new PES surface change treatments (Table 3).

Treatments 12 and 13 were performed to determine the possible influence of alkaline hydrolysis on the surface modifications and GL fixation. The moisture content percentage was increased by approximately 30% in 10 to 13% alkaline media. The PES moisture gain was also evaluated after the treatments and after rinsing, and limited differences were observed between the treatments. The moisture gain due to the alkaline media after rinsing to determine GL fixation was low and remained at approximately 10% in the two cases.

Microwave irradiation was also applied in treatments 14 to 17 with 50% GL over the fiber weight and treatments 18 to 21 with 100% glycerol over the fiber weight in alkaline or neutral media at 2 min 300 W or 5 min 500 W, with all of them performed at a bath ratio of 1/15. As expected, the moisture content always increased with the amount of GL in treatments 18–21 and with higher irradiation conditions in treatments 19 and 21. However, the presence of the alkali solution in treatments 18 and 19 did not seem to enhance PES moisture. Moreover, the moisture content was maintained at approximately 20% in treatments 20 and 21 (without alkali) after rinsing.

Therefore, certain treatments were studied in depth to determine the best method of increasing the moisture content and identify their water sorption and permeability properties and comfort performance. Treatment 11 was studied due to the important moisture increase despite the lack of regularity of the fabric and the prolonged treatment time. The results indicated that bath ratio of 1/15 and 100% glycerol are good conditions to achieve moisture gain. Using bath exhaustion, treatment 12 in a neutral medium and treatment 13 in an alkaline medium led to the best moisture contents. Using microwave irradiation, treatment 21 in a neutral medium presented much better results and reached more than 2% moisture of the fabric after rinsing. Therefore, the moisture at 65% RH was determined by gravimetry and the wicking test; moisture at 95% RH and apparent diffusion was determined by Dynamic Vapor Sorption; water vapor resistance was determined by means of the Permetest instrument. Negative ions were detected in the PES treated with GL at the optimized experimental conditions and compared with the values of the PES, CO and WO fibers (Table 1) to determine the improved humidity permeability and comfort properties (Table 4).

The obtained results indicate an increase in moisture of more than 10% at 65% RH in all cases after the GL treatment, and the moisture after rinsing was maintained at more than 1%. Treatments 11, 13 and 21 presented the highest (and similar) increases in moisture at between 12.3 and 13.3%, and it should be noted that the highest moisture obtained for treatment 21 using microwaves was maintained at a value greater than 2% after rinsing. The moisture percentage at 95% RH using DVS followed the same tendency as that at 65% RH, although as expected, the values were higher. Treatment 12 presented the lowest value, followed by treatments 21 and 11, with the NaOH treatment having the highest moisture content. Moisture wicking was also increased in all treatments; however, the main difference from moisture absorption was that moisture was not lost after rinsing. Moisture wicking seems more related to the experimental conditions (T, energy, time, etc.) than to the presence of GL. The best results were obtained with treatment 13. All treatments with GL promoted a clear increase in negative ions from 1 PES to between 10 and 12 anions/cm^3^, with treatment 13 having the highest value, which indicates better comfort. The diffusion of water through the textiles evaluated by DVS presented an important decline (from 60 to 75%), and for all GL-treated fibers, this decrease was related to an increase in Ret (from 20% to 45%). While treatments 11, 13 and 21 presented very similar low water diffusion values, treatments 11 and 21 presented similar high vapor resistance. Moreover, treatment 12 had the lowest moisture percentage increase as well as high water diffusion and high permeability.

The R_et_ values increased after all GL treatments, although the values of approximately 2 indicated good transpiration; thus, the treated PES fabrics have the capacity to expel moisture/sweat from the interior to the exterior. The high values of wicking demonstrated the fabric’s ability to move moisture away from the body, thus preventing the fabric from becoming saturated. These values are related to R_et_. Therefore, GL gives sufficient hydrophilicity to the fabric to provide a feeling of comfort without impeding breathability.

In summary, although treatment 13 with alkali promoted the highest moisture increase under the highest moisture wicking and highest negative ion production conditions, treatments 11 and 21 presented high moisture contents with low water diffusion and the highest water vapor resistance, with treatment 21 having the highest glycerol retention after rinsing.

**Figure 1:**
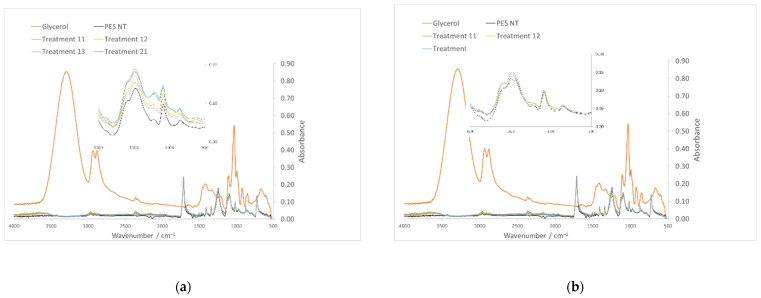
ATR-FTIR spectra of PES, GL, nontreated PES and GL-treated PES fibers (Table 3) before (**a**) and after rinsing (**b**).

The ATR-FTIR spectra of GL, nontreated PES and GL-treated PES fibers (Table 4) were obtained to determine the presence of GL on the fibers. The spectra of the treated fibers before and after rinsing are visualized in Figure 1a,b, respectively. The GL peaks at 3300 and 1040 cm^−1^ corresponded to the associated OH stretching and C–O stretching. These bands can be clearly seen in the GL-treated textiles before rinsing but are less obvious after rinsing, and they were more prominent for treatments 11 and 13. After rinsing, only a small increase in a broad peak at higher frequencies ≈ 3600 cm^−1^ was observed, which could correspond to free OH stretching resulting from the small residual glycerol or NaOH content as well as the slight increase in moisture absorption in some of the cases.

**Figure 2:**
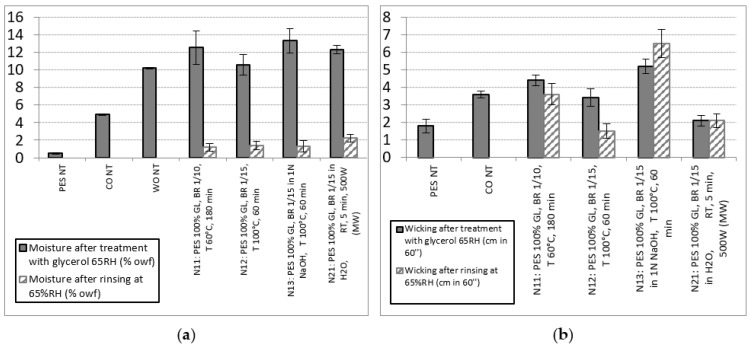
Moisture content at 65% RH (**a**) and wicking (**b**) of PES, CO and WO and the GL-treated PES fibers and after rinsing.

**Figure 3:**
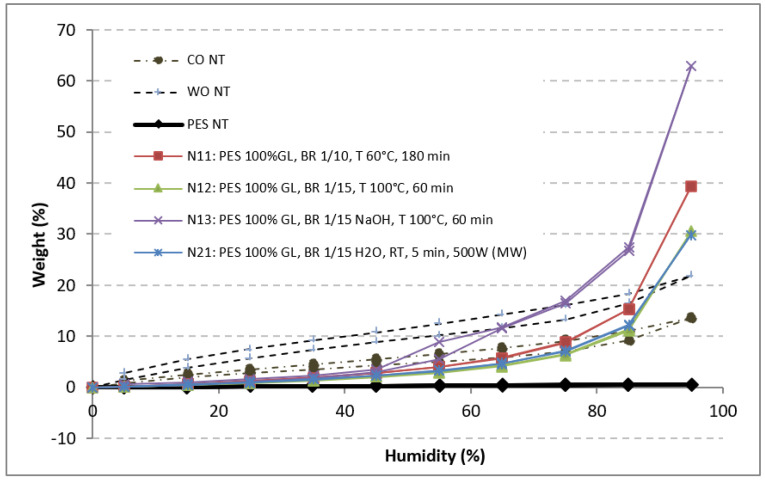
Water absorption/desorption of PES, CO and WO (Table 1) and the GL-treated PES fibers (Table 4).

The main properties of the GL-treated fibers were also compared with those of the natural fibers CO and WO. The moisture values at 65% RH and the wicking and water absorption/desorption at different% RH by DVS can be easily visualized and compared with non-treated PES and CO or WO (in some cases) in Figure 2 and Figure 3.

The moisture in the PES fibers treated with GL significantly increased and was higher than the values obtained for CO and even WO. Although this moisture content decreased after rinsing, it was maintained at values greater than 1 for all treatments and greater than 2 for treatment 21 with the use of microwaves (Figure 2). The moisture wicking values demonstrate a favorable increase mainly due to the heating bath exhaustion treatments prior to the microwave treatments, and these effects were independent of the amount of glycerol absorbed.

The percentage of absorption and desorption of water at different RHs obtained based on the DVS of PES-, CO- and WO- and GL-treated fibers is graphically shown in Figure 3. Plain PES generally did not absorb/desorb water at all RHs (less than 1%), and WO presented a sigmoidal shape, with a maximum moisture absorption of 14 and 22%, with a marked hysteresis between desorption and absorption, which is common in natural fibers. The GL-treated fibers presented higher moisture than PES, which drastically increased at high RH. However, the shape and hysteresis observed in the sample treated with NaOH (treatment 13) demonstrated superficial dissolution of PES [34]. The rest of the PES treatments that did not exhibit hysteresis showed that the moisture did not modify the PES sufficiently to reach the behavior of natural fibers (CO and WO), where clear hysteresis occurred.

The apparent diffusion results indicated a decrease in the speed of water diffusion for all GL-treated fibers, although the results did not reach the values of either CO or WO. However, the R_et_ values reached levels similar to those for CO. In addition, a similar trend occurred for the negative ions (Table 4). PES without treatment barely provided any ions (1 anions/cm^3^), whereas the samples treated with GL slightly increased the number of ions, which reached approximately 10 an ions/cm^3^. This increase was even higher for PES treated with NaOH. The electrical conductivity of water is well-known. Therefore, this increase in ion generation can be directly related to water absorption and the increase in ion transfer can be directly related to the increase in moisture retention.

## 4. Conclusions

In this study the main focus was PES modification to increase the moisture capacity with the consequent improvement in the user’s well-being.

Polyester fiber itself has a very low moisture content and thus a very low comfort level. Based on the hydroscopic properties of GL and its use in textile processing as a solvent, thickener and dispersant, it was applied to promote the hydrophilicity of PES. To improve the fixation of GL, the PES fabric was pretreated with an alkaline solution and microwave irradiation. PES GL-treated samples were characterized in terms of their% moisture, negative ions, water diffusion and water vapor resistance. 

The moisture content was determined for GL-treated PES fabric and the results showed that decreasing the bath ratio and increasing the time and temperature led to a moisture gain of almost 14%, which can be easily related to the weight increase of the fabric. To increase the linkage between the PES fabric and GL, the surface characteristics of the fiber were altered by pretreatment with a strong alkaline solution or microwave irradiation. Alkaline treatment 13 (PES 100% GL, BR 1/15 in 1N NaOH, T 100 °C, 60 min) led to the highest moisture increase after treatment (13.3%) and highest negative ion production (12 anions/cm^3^), and treatments 11 (PES 100% GL, BR 1/10, T 60 °C, 180 min) and 21 (PES 100% GL, BR 1/15 in H_2_O, RT, 5 min, 500 W) presented a high moisture contents with low water diffusion and the highest water vapor resistance. Moreover, treatment 21 had higher GL retention after rinsing. These results were related to the presence of glycerol in the treated fibers based on ATR-FTIR. The main properties of the GL-treated fibers were also compared with those of the natural fibers CO and WO. The moisture in the PES fibers treated with GL significantly increased and was higher than that of CO and even WO. Although this humidity decreased after several rinses, it is worth noting that it was maintained at values greater than 1 in all treatments and greater than 2 in treatment 21 (PES 100% GL, BR 1/15 in H_2_O, RT, 5 min, 500 W) with the use of microwaves. The apparent diffusion results indicate a decrease in the speed of water diffusion for all GL-treated fibers; in addition, the water vapor resistance values reached levels similar to those for CO. The increase in comfort can be determined by the negative ion value, which reached values of approximately 10 to 12 (anions/cm^3^).

So, the results show that treating PES fabric with GL obtained in a clear improvement in the moisture retention capacity promoting the comfort sensation, as demonstrated by the R_et_ and negative ion analyses. The surface-related properties of PES fabric were modified by an alkali treatment and/or microwave irradiation prior to the GL treatment to improve wash fastness, although the obtained results were not as good as desired. Therefore, other strategies, such as low-temperature plasma pretreatment with oxidative gases, will be applied in the future to increase the linkage between PES fabric and glycerol and therefore achieve the desired comfort properties similar to that of natural fibers.

## Figures and Tables

**Table 1:** Moisture at 65% RH, Moisture at 95% RH and Apparent Diffusion by DVS, R_et_ and Negative Ions (NIs) for non-treated fabrics (NT).

Sample	Moist. Content at 65% RH (% owf)	DVS		R_et_ (Pa × m^2^ × W^−1^)	NIs (anions/cm^3^)
		Moist 95% RH (%)	D_a_ (min^−1^ × 10^3^)		
**PES NT**	0.5 ± 0.1	0.59 ± 0.06	0.90 ± 0.03	1.71 ± 0.06	0.6 ± 0.4
**CO NT**	4.9 ± 0.1	13.67 ± 0.16	0.08 ± 0.01	2.20 ± 0.25	27.0 ± 4.2
**WO NT**	10.2 ± 0.1	21.80 ± 0.22	0.04 ± 0.01	8.88 ± 0.50	14.4 ± 1.5

**Table 2:** Moisture at 65% RH and increase in weight of PES/GL treatments before and after rinsing. Bath exhaustion of GL solution at different concentrations (3%, 50% and 100% owf), different bath ratio BR 1/15, 1/10, different temperatures T, 40°C, 60°C at 60 and 180 min.

N	Sample/Treatment	Moisture at 65% RH (% owf)	Weight Increase after Treat. (% owf)	Weight Increase after Treat. and after Rinsing (% owf)
	Non Treated PES (PES NT)	0.5 ± 0.1	-	-
1	PES 3% GL, BR 1/15, T 40 °C, 60 min	1.0 ± 0.1	0.4 ± 0.02	0 ± 0.01
2	PES 50% GL, BR 1/15, T 40 °C, 60 min	4.5 ± 0.50	4.5 ± 0.3	0.5 ± 0.1
3	PES 100% GL, BR 1/15, T 40 °C, 60 min	9.2 ± 0.78	9.8 ± 0.81	1.9 ± 0.06
4	PES 50% GL, BR 1/10, T 40 °C, 60 min	6.2 ± 0.05	5.8 ± 0.09	0.9 ± 0.02
5	PES 100% GL, BR 1/10, T 40 °C, 60 min	11.3 ± 0.72	14.5 ± 0.2	2.8 ± 0.08
6	PES 50% GL, BR 1/10, T 40 °C, 180 min	8.2 ± 0.33	8.4 ± 0.4	4.3 ± 0.1
7	PES 100% GL, BR 1/10, T 40 °C, 180 min	12.0 ± 0.37	14.8 ± 0.6	7.6 ± 0.2
8	PES 50% GL, BR 1/10, T 60 °C, 60 min	9.6 ± 3.33	9.6 ± 0.9	1.0 ± 0.02
9	PES 100% GL, BR 1/10, T 60 °C, 60 min	11.5 ± 1.17	14.7 ± 0.88	4.9 ± 0.3
10	PES 50% GL, BR 1/10, T 60 °C, 180 min	11.9 ± 0.12	10.8 ± 0.3	4.2 ± 0.2
11	PES 100% GL, BR 1/10, T 60 °C, 180 min	12.9 ± 0.20	17.2 ± 0.98	7.6 ± 0.4
12	PES 100% GL, BR 1/15, T 100 °C, 60 min	10.9 ± 0.65	11.1 ± 0.7	4.1 ± 0.4

N = Treatment number

**Table 3:** Moisture content of the PES/GL treatments before and after rinsing. Bath exhaustion of 100% GL owf at a 1/15 bath ratio at 100 °C, t = 60 min in neutral or alkaline conditions and with microwave irradiation (MW) of the GL solution at different concentrations, power and treatment times.

N	Sample/Treatment	Moisture at 65%RH (% owf)	Moisture after Rinsing at 65%RH (% owf)
	Non Treated PES (PES NT)	0.5 ± 0.1	-
11	PES 100% GL, BR 1/10, T 60 °C, 180 min	12.6 ± 0.2	1.2 ± 0.3
12	PES 100% GL, BR 1/15, T 100 °C, 60 min	10.1 ± 1.0	1.71 ± 0.3
13	PES 100% GL, BR 1/15 NaOH, T 100 °C, 60 min	13.5 ± 1.2	1.51 ± 0.5
14	PES 50%GL, BR 1/15 NaOH, RT, 2 min, 300 W (MW)	6.4 ± 0.9	0.84 ± 0.4
15	PES 50%GL, BR 1/15 NaOH, RT, 5 min, 500 W (MW)	8.8 ± 0.9	1.68 ± 0.3
16	PES 50% GL, BR 1/15 H_2_O, RT, 2 min, 300 W (MW)	4.6 ± 0.4	1.68 ± 0.2
17	PES 50% GL, BR 1/15 H_2_O, RT, 5 min, 500 W (MW)	7.6 ± 0.6	1.26 ± 0.7
18	PES 100% GL, BR 1/15 NaOH, RT, 2 min, 300 W (MW)	10.2 ± 0.6	2.19 ± 0.9
19	PES 100% GL, BR 1/15 NaOH, RT, 5 min, 500 W (MW)	13.0 ± 0.5	3.42 ± 0.5
20	PES 100% GL, BR 1/15 H_2_O, RT, 2 min, 300 W (MW)	11.8 ± 0.7	2.40 ± 0.1
21	PES 100% GL, BR 1/15 H_2_O, RT, 5 min, 500 W (MW)	12.4 ± 0.9	2.25 ± 0.9

N = Treatment number

**Table 4:** Moisture at 95%RH and Apparent Diffusion (D_a_) by Dynamic Vapor Sorption, Water Vapor Resistance (R_et_) and Negative Ions (NI) of the different textiles, and PES treated with GL at the optimized experimental conditions.

N	Sample	DVS		R_et_ (Pa × m^2^ × w^−1^)	NI (anions/cm^3^)
		Moist 95RH (%)	D_a_ (min^−1^ × 10^3^)		
	Non Treated PES (PES NT)	0.6	0.90	1.71 ± 0.06	0.6 ± 0.4
**11**	PES 100% GL, BR 1/10, T 60 °C, 180 min	36.3	0.26	2.48 ± 0.67	10.3 ± 1.5
**12**	PES 100% GL, BR 1/15, T 100 °C, 60 min	22.5	0.37	2.01 ± 0.32	9.9 ± 0.8
**13**	PES 100% GL, BR 1/15 in 1N NaOH, T 100 °C, 60 min	53.3	0.22	2.18 ± 0.18	12.0 ± 0.5
**21**	PES 100% GL, BR 1/15 in H_2_O, RT, 5 min, 500 W	34.4	0.26	2.33 ± 0.59	9.6 ± 0.7

N = Treatment number

## Data Availability

Not applicable.
